# An effectiveness evaluation of a community-based course for medical students: a randomized controlled trial in the teaching of epidemiology

**DOI:** 10.1186/s12909-023-04787-z

**Published:** 2023-10-27

**Authors:** Yongming Zhang, Ting Huang, Mengling Tang, Lin Meng, Xiaolu Wu, Kun Chen

**Affiliations:** 1 Department of Medical Education, The Fourth Affiliated Hospital, Zhejiang University School of Medicine, Yiwu, 322000 China; 2 Department of Ophthalmology, The Sir Run Run Shaw Hospital, Zhejiang University School of Medicine, Hangzhou, 310016 China; 3 Department of Public Health, The Fourth Affiliated Hospital, Zhejiang University School of Medicine, Hangzhou, 310058 China; 4 Department of Public Health, The Second Affiliated Hospital, Zhejiang University School of Medicine, Hangzhou, 310058 China

**Keywords:** Kern's six-step approach, Community-based course, Epidemiology, Medical education

## Abstract

**Background:**

Epidemiology is considered to be the fundamental science of public health and plays an important role in clinical competence and professional development. The objective of this study is to evaluate the effectiveness of a short-term course for the teaching of epidemiology, which was designed as a community-based class for medical students.

**Method:**

This course was designed according to Kern’s six-step approach to curriculum development. A total of 75 undergraduates were recruited. Forty-one students were assigned to an experimental group engaged in theoretical teaching and practical courses, while 34 students were assigned to the control group only taking theoretical courses. All participants were asked to complete a pre- and post-course survey and to take a test after completing the course. The scores between the experimental and control groups were compared using the Wilcoxon test.

**Result:**

The experimental group showed significantly higher self-assessment scores in course understanding (*p* = 0.0126) and clinical practice skills (*p* = 0.0005) after completing the course, while no significant difference was observed in the control group. In addition, students in the experimental group reported significantly higher interest (*p* = 0.0015), stronger learning motivation (*p* = 0.0113) and a better mastery of epidemiology (*p* = 0.0167) after completing the course than those in the control group. However, test scores (*p* = 0.0859) and pass rates (*p* = 0.1755) demonstrated no statistical significance between the two groups.

**Conclusion:**

The short-term practical course in epidemiology exerted significantly positive effects on the improvement of student learning enthusiasm, course understanding and clinical practice skills. These findings provide new ideas and statistical evidence for the development of epidemiological instruction. Future studies should explore how to more widely and optimally apply community-based courses to the teaching of epidemiology.

**Supplementary Information:**

The online version contains supplementary material available at 10.1186/s12909-023-04787-z.

## Introduction

Epidemiology, the key course in public health programs, is a critical component of medical-related undergraduate curricula and is defined as being fundamental to population health science [1–4]. This discipline is of paramount importance for cultivating medical students’ clinical practice abilities, which include ethical reasoning, critical thinking, quantitative literacy, causal inference and teamwork [5]. The need, as an educator, is to optimize the teaching contents and approaches taken in epidemiology education and to ensure that students have a strong command of epidemiological knowledge and the ability to apply probabilistic thinking to scientific research and clinical decision-making.

During the past few years, epidemiology has expanded from the study of infectious diseases to encompass health-related outcomes, including the exposure factors of various acute and chronic diseases that have been observed through clinical diagnoses [6]. In fact, many published epidemiological studies have exerted critical clinical impacts on efforts to increase awareness of the value of epidemiology because these research findings were often translated into guidelines, hospital policies and standardized clinical decisions [7, 8]. For instance, a prospective cohort study [9] provided robust evidence of the causal link between obesity and the incidence of certain cancers, thus offering a clinical basis for weight control. In addition, evidence has shown that clinicians are unfamiliar with health statistics and research designs [10, 11], which are important parts of the epidemiological investigation. Without these capacities, such studies may fail to transform “real-world data” into improved clinical care and health outcomes [10, 12].

In 1999, the Association of American Medical Colleges (AAMC) stated that the best method for medical teaching was teaching through examples and experiences, rather than simply relying on theoretical courses [13]. To date, most medical colleges still adopt the traditional education mode, which emphasizes theoretical teaching and lacks practical instruction in epidemiology [14]. Adopting this mode can result in passive learning and weak capabilities among medical undergraduates regarding clinical problem-solving and scientific research. To solve this problem, we designed a short-term practical course and applied it to the clinical teaching of epidemiology. This study aims to evaluate the effectiveness of this community-based course for medical students.

## Method

### Curriculum design

The practical course of epidemiology was developed based on the six-step approach of curriculum development for medical education [15], which includes problem identification and general needs assessment, targeted needs assessment, measurable goals, educational strategy, implementation, feedback and evaluation. Before the study, we conducted a forum to collect undergraduate opinions on epidemiology instruction, and 70 (93.33%) students expressed a demand for practical and clinically relevant teaching methods.

### Curriculum settings

The participants were divided into the experimental group and the control group using the method of random number table of the simple randomization. The members of both two groups were required to attend theoretical courses. The study included four theoretical classes in total, lasting for a month and each week, students took a theoretical course. A theoretical course took approximately 4 h. The first theoretical course was the introduction to clinical epidemiology, which introduced the history and development of clinical epidemiology. The second course consisted of an overview of common epidemiological research methods, and students learned about the design, advantages and disadvantages of various epidemiological methods, including cross-sectional studies, case‒control studies, cohort studies, and experimental studies. The third theoretical course focused on practical epidemiological research and included statistical description and analysis, providing students with a clear understanding of data collection, data entry, statistical description and statistical inference. Investigator training was a prerequisite for the epidemiological practice course, and we set it as the last course in the theory series. The course contained how to be an investigator from the beginning of proposing research questions, research design and implementation, data collection, statistic analysis and result discussion, as well as communication skills, data-gathering techniques, which were honestly needed in the actual research process. On the one hand, this allowed students to review the content of the previous lessons through the questionnaires used in practice. On the other hand, it ensured that students could complete the practice course with high quality.

The epidemiological practice course conducted in community was only applicable to the experimental group and the community practice course was conducted after each theoretical course in the last two weeks. All the students engaged with every link in a cohort study that was specifically designed for this course. When students in the experimental group conducted the practical course in the community, students in the control group were arranged self-learning in the cohort study cases. The specified cohort study began with a literature review, then a research purpose was proposed and the research contents were determined. The next step involved research design and implementation, which included determining the study method, exposure factors, health outcomes, study site, study population and sample size. Next, students participated in data collection, and in this section, medical ethics and data authenticity were also considered. Students conduct statistical analysis after the completion of data collection. Finally, they discuss the results and possible biases of the study. The practical course conducted in the community was in conjunction with a field test that aimed to develop a more specific health literacy scale for elderly Chinese individuals [16].

### Study participants

Undergraduates who entered the Fourth Affiliated Hospital of Zhejiang University for internship were recruited in June 2021, then randomly divided into summer term and fall term for internship. These students came from six different universities and were informed that there would be a pre- and post-survey conducted to self-rate their levels of competency, perception, expectation and satisfaction with the epidemiological courses, as well as a test after completion of the class to assess the course effectiveness.

A total of 75 medical students participated in the designed course, the self-evaluation and the follow-up test. Forty-one students who began their internship in the summer term were divided into the experimental group (taking theoretical courses integrated with a practical course), and 34 students who began their internship in the autumn term were assigned to the control group, and thus only took the theoretical courses. All the available data collected from the 75 interns were included in the analysis.

### Data collection

Before conducting the study, students received explanations regarding the courses, surveys and tests that they would participate in and provided confirmation of their agreement to the data collection and processing methods. Data collection included the self-assessments taken before and after the epidemiological course and the follow-up test scores. Basic information, including age, sex, university, major and prior experience in the study of epidemiology, was also collected through the survey. All data were collected on-site.

### Effect evaluation

Medical students were asked to complete a self-rated survey both before and after taking courses using a 5-point Likert scale (1 = strongly disagree to 5 = strongly agree). The survey items included student perceptions towards epidemiological courses, course understanding and clinical practice competencies. Comparisons were conducted of the average survey scores between the different groups and those of the same group from before and after taking the epidemiological course. Furthermore, we tested all participants with theoretical and case-related single-choice questions after the course. The test questions were related to epidemiological characteristics, participants, sampling methods, statistical methods, statistic description, research bias and the application issues of these contents in practical cases. Test score of theoretical part, case analysis part and total score between the experimental and control groups were compared.

### Statistical analysis

The ages of the participants are presented as the mean ± standard deviation (SD), and other basic information, such as sex and learning experience, was tested with the chi-square test and displayed as frequencies and percentages. The survey responses and the final test scores were skewed, so scores are presented as the median ± interquartile range (IQR), and the Mann‒Whitney U test was applied to compare the survey responses and test scores between the two groups. For the comparison of pre- and post-course survey scores in the same group, the Wilcoxon signed-rank test was applied. To adjust for the confounding factors like age and sex, liner regression analysis was conducted to figure out the association between test scores and the practical course. We set an alpha level of *p* < 0.05 as representative of statistical significance. All analyses were performed with R software (version 4.0.4).

## Result

A total of 75 medical students were included in this study, with 41 in the experimental group, and 34 in the control group. The participants included students from six different schools, but 55 (73.33%) students came from Zhejiang University The average ages (standard deviation) of students in the experimental and control groups were 22.51 (0.93) years and 22.82 (0.58) years, respectively. Among all participants, the experimental group included 15 (20.0%) males and 26 (34.67%) females, while the control group included 13 (17.33%) males and 21 (28.0%) females. The survey results showed that all students had experience with the theoretical teaching of epidemiology, while only 2.67% of the students in the experimental group and 5.33% of those in the control group had ever taken a practical course in epidemiology.

The survey responses of the two groups from before and after class are shown in Supplementary Table 1. In the experimental group, only 15.0% of the participants claimed that they understood the epidemiological course before taking the class, while 65.85% of the students claimed attaining such an understanding after the course. The percentage of participants who considered themselves to be good at utilizing epidemiological skills increased from 2.50% in the pre-course survey to 19.51% in the post-survey. After completing the course, more students (from 65.00% to 82.93%) expressed an interest in the epidemiological course, and 95.12% of the students regarded the mastery of epidemiological skills as necessary and helpful for clinicians in medical scientific research.

The percentage of students in the control group who claimed to understand the course increased from 38.24% to 70.59%, and the number of students who thought that practice was more helpful than theory also increased from 15 (50.0%) to 24 (70.59%). The percentage of students in the experimental group who were eager to participate in this course increased slightly from 72.50% to 80.49%, while that percentage remained unchanged (47.06%) in the control group. Overall, students in both groups showed greater agreement with positive perceptions, a deeper course understanding and stronger clinical practice skills after completing the course.

As shown in Table 1, after the integration course, the self-assessment score of undergraduates in the experimental group was significantly higher for course understanding (*p* = 0.0126) and clinical practice competency (*p* = 0.0005), but the perceptions of students towards the course showed nonsignificant changes. These changes specifically reflected the fact that students had a better understanding of course content (*p* < 0.0001) and had become better at using epidemiological methods to solve clinical problems (*p* = 0.0001). However, in the control group, no significant difference was observed across all self-assessment dimensions.

**Table 1:** Comparison of the pre- and post-course survey scores in each group

Survey questions		Experimental group (*n* = 40)			Control group (*n* = 34)		
		pre-course	post-course	*p* value	pre-course	post-course	*p* value
Perception of the epidemiological course		3.83 ± 0.76	3.92 ± 0.74	0.4209	3.52 ± 0.85	3.58 ± 0.93	0.4685
Q1	I am interested in this course	3.83 ± 0.84	3.93 ± 0.69	0.5358	3.21 ± 0.91	3.38 ± 0.85	0.4379
Q2	I truly want to participate in this practical course	3.88 ± 0.79	3.95 ± 0.75	0.5587	3.53 ± 0.86	3.38 ± 1.07	0.5261
Q3	I am looking forwards to/satisfied with this course	3.80 ± 0.65	3.87 ± 0.81	0.8771	3.87 ± 0.63	4.07 ± 0.60	0.1270
Course understanding		3.21 ± 1.01	3.53 ± 0.90	**0.0126**	3.18 ± 0.89	3.37 ± 1.02	0.1705
Q4	I comprehend the epidemiological course contents	2.60 ± 0.96	3.58 ± 0.78	** < 0.0001**	3.21 ± 0.95	3.65 ± 0.85	0.0896
Q5	I think this course is easy to master	3.18 ± 0.84	3.18 ± 0.90	0.9139	2.74 ± 0.71	2.68 ± 0.98	0.6128
Q6	I think practice is more helpful than theory in the teaching of epidemiology	3.85 ± 0.83	3.83 ± 0.90	0.8899	3.59 ± 0.82	3.79 ± 0.88	0.2273
Clinical practice competency		3.69 ± 1.16	3.96 ± 0.89	**0.0005**	3.76 ± 0.91	3.78 ± 0.94	0.5870
Q7	I think this course is helpful for medical scientific research	4.35 ± 0.53	4.35 ± 0.58	0.9425	4.18 ± 0.58	4.26 ± 0.62	0.4883
Q8	I am good at utilizing epidemiological methods	2.08 ± 0.83	2.88 ± 0.79	**0.0001**	2.74 ± 0.83	2.74 ± 0.93	0.9623
Q9	I think it is necessary for clinicians to be trained in this course	4.13 ± 0.72	4.28 ± 0.55	0.2793	4.06 ± 0.69	4.00 ± 0.78	0.8357
Q10	I think it is helpful for clinicians to master epidemiological skills	4.20 ± 0.65	4.33 ± 0.57	0.3606	4.06 ± 0.65	4.12 ± 0.48	0.6695

Data are presented as the mean ± standard deviation (SD); the *p* value was calculated based on the Wilcoxon signed-rank test

Before the course, the average survey scores of students in the experimental group were 2.60 ± 0.96 in comprehending the course contents and 2.08 ± 0.83 in the use of epidemiological methods, which are both far lower than the 3.21 ± 0.95 and 2.74 ± 0.83 found in the control group. The survey results also showed significant differences in these two questions, but these differences did not appear after completion of the course. As shown in Table 2, significantly higher scores in perceptions towards epidemiological courses were observed in the experimental group both in pre- (*p* = 0.0068) and post-course (*p* = 0.0032) surveys. Throughout the course, students in the experimental group exhibited more positive attitudes towards epidemiological learning, showing greater interest in the course (*p* = 0.0015) and a stronger desire to participate in it (*p* = 0.0113). Furthermore, although the dimension of course understanding showed nonsignificant change, students in the experimental group reported thinking that the course was easier to master (*p* = 0.0167).

**Table 2:** Comparison of the self-rated survey scores between the experimental and control groups

Statement numbers		Pre-course			Post-course		
		Experimental group (*n* = 40)	Control group (*n* = 34)	*p* value	Experimental group (*n* = 41)	Control group (*n* = 34)	*p* value
Perceptions		3.83 ± 0.76	3.52 ± 0.85	**0.0068**	3.92 ± 0.73	3.58 ± 0.93	**0.0032**
Q1	n (missing)	40 (0)	34 (0)	**0.0054**	41 (0)	34 (0)	**0.0015**
	Mean ± SD	3.83 ± 0.84	3.21 ± 0.91		3.93 ± 0.69	3.38 ± 0.85	
	median (IQR)	4 (3, 4)	3 (3, 4)		4 (4, 4)	3 (3, 4)	
Q2	n (missing)	40 (0)	34 (0)	0.0602	41 (0)	34 (0)	**0.0113**
	Mean ± SD	3.88 ± 0.79	3.53 ± 0.86		3.95 ± 0.74	3.38 ± 1.07	
	median (IQR)	4 (3, 4)	3 (3, 4)		4 (4, 4)	3 (3, 4)	
Q3	n (missing)	40 (0)	30 (4)	0.6535	32 (9)	28 (6)	0.3850
	Mean ± SD	3.80 ± 0.65	3.87 ± 0.63		3.88 ± 0.79	4.07 ± 0.60	
	median (IQR)	4 (3, 4)	4 (3.25, 4)		4 (3.75, 4)	4 (4, 4)	
Course understanding		3.21 ± 1.01	3.18 ± 0.89	0.7080	3.53 ± 0.89	3.37 ± 1.02	0.3279
Q4	n (missing)	40 (0)	34 (0)	**0.0060**	41 (0)	34 (0)	0.6340
	Mean ± SD	2.60 ± 0.96	3.21 ± 0.95		3.59 ± 0.77	3.65 ± 0.85	
	median (IQR)	3 (2, 3)	3 (3, 4)		4 (3, 4)	4 (3, 4)	
Q5	n (missing)	40 (0)	34 (0)	**0.0113**	41 (0)	34 (0)	**0.0167**
	Mean ± SD	3.18 ± 0.84	2.74 ± 0.71		3.17 ± 0.89	2.68 ± 0.98	
	median (IQR)	3 (3, 4)	3 (2, 3)		3 (2, 4)	2 (2, 3)	
Q6	n (missing)	40 (0)	34 (0)	0.1541	41 (0)	34 (0)	0.9455
	Mean ± SD	3.85 ± 0.83	3.59 ± 0.82		3.83 ± 0.89	3.79 ± 0.88	
	median (IQR)	4 (3, 4)	3.5 (3, 4)		4 (3, 4)	4 (3, 4)	
Clinical practice competency		3.69 ± 1.16	3.76 ± 0.91	0.9059	3.95 ± 0.88	3.78 ± 0.94	0.1250
Q7	n (missing)	40 (0)	34 (0)	0.2052	41 (0)	34 (0)	0.6195
	Mean ± SD	4.35 ± 0.53	4.18 ± 0.58		4.34 ± 0.57	4.26 ± 0.62	
	median (IQR)	4 (4, 5)	4 (4, 4.75)		4 (4, 5)	4 (4, 5)	
Q8	n (missing)	40 (0)	34 (0)	**0.0019**	41 (0)	34 (0)	0.3461
	Mean ± SD	2.08 ± 0.83	2.74 ± 0.83		2.88 ± 0.78	2.74 ± 0.93	
	median (IQR)	2 (1, 3)	3 (2, 3)		3 (2, 3)	3 (2, 3)	
Q9	n (missing)	40 (0)	34 (0)	0.7366	41 (0)	34 (0)	0.1287
	Mean ± SD	4.13 ± 0.72	4.06 ± 0.69		4.27 ± 0.55	4.00 ± 0.78	
	median (IQR)	4 (4, 5)	4 (4, 4)		4 (4, 5)	4 (4, 4)	
Q10	n (missing)	40 (0)	34 (0)	0.3854	41 (0)	34 (0)	0.0951
	Mean ± SD	4.20 ± 0.65	4.06 ± 0.65		4.32 ± 0.57	4.12 ± 0.48	
	median (IQR)	4 (4, 5)	4 (4, 4)		4 (4, 5)	4 (4, 4)	

*SD *is the standard deviation, *IQR* is the interquartile range; and the *p* value was calculated based on the Mann‒Whitney U test

In the final test, the average test scores in the experimental group and control group were 47.85 ± 6.50 and 46.18 ± 5.71 for the theoretical part and 31.02 ± 7.20 and 29.58 ± 7.61 for the case analysis part, respectively (Table 3). In this study, we set a pass score of 80 out of 100. The pass rate in the experimental group was 60.98%, while only 42.42% of students in the control group passed the class. Although the average score and pass rate increased among the experimental group, the statistical analysis showed that there were no significant differences between the two groups (*p* = 0.0859 and *p* = 0.1755, respectively). Linear regression analysis was also conducted to figure out the association between test scores and the practical course, which taking the confounding factors adjustment into account. As shown in Table 4, the coefficient β indicated that the total scores in the experimental group were 1.738 times higher than those of the control group. The scores of participants who accepted the practical course were 1.328 times and 0.410 times higher than those not received the practical course in the theoretical part and case-analysis part respectively, but the *p* value (*p* = 0.430 and 0.841) identified the association was insignificant. Supplementary Table 2 provided more analysis results and made adjustments for the baseline group differences of the three dimensions. The results were consistent with the statistical results shown in Table 3.

**Table 3:** Comparison of the test scores and pass rate between the experimental and control groups

Section		Experimental group (*n* = 41)	Control group (*n* = 33)	*p* value
Theoretical part	Mean ± SD	47.85 ± 6.50	46.18 ± 5.71	0.1958
	median (IQR)	48 (48, 54)	48 (42, 48)	
Case analysis part	Mean ± SD	31.02 ± 7.20	29.58 ± 7.61	0.4227
	median (IQR)	32 (24, 40)	32 (24, 32)	
Total score	Mean ± SD	78.88 ± 9.93	75.76 ± 8.69	0.0859
	median (IQR)	80 (72, 86)	74 (70, 82)	
Pass score (> = 80)	pass rate (%)	60.98	42.42	0.1755

*SD* is the standard deviation, *IQR* is the interquartile range; and the *p* value was calculated based on the Mann‒Whitney U test

**Table 4:** The association between test score and the community practical course

		Model 1		Model 2		Model 3		Model 4	
		coefficient β (95% CI)	*p* value	coefficient β (95% CI)	*p* value	coefficient β (95% CI)	*p* value	coefficient β (95% CI)	*p* value
Theoretical part	Control	(Reference)		(Reference)		(Reference)		(Reference)	
	Experimental	1.253 (-1.756, 4.261)	0.417	1.758 (-1.400, 4.916)	0.279	1.869 (-1.046, 4.783)	0.213	1.328 (-1.951, 4.606)	0.430
Case-analysis part	Control	(Reference)		(Reference)		(Reference)		(Reference)	
	Experimental	1.247 (-2.463, 4.957)	0.512	0.471 (-3.360, 4.302)	0.810	0.928 (-2.611, 4.466)	0.609	0.410 (-3.587, 4.407)	0.841
Total score	Control	(Reference)		(Reference)		(Reference)		(Reference)	
	Experimental	2.499 (-2.248, 7.246)	0.306	2.230 (-2.693, 7.153)	0.378	2.797 (-1.755, 7.348)	0.233	1.738 (-3.415, 6.891)	0.511

Test scores of students in the control group was set as the reference and test scores included scores of theoretical part, scores of case-analysis part and total score

Model 1 adjusted for age, gender and the self-rated score of question 1 before the course

Model 2 adjusted for age, gender and the self-rated score of question 4, 5 before the course

Model 3 adjusted for age, gender and the self-rated score of question 8 before the course

Model 4 adjusted for age, gender and the self-rated score of question 1, 4, 5, 8 before the course

## Discussion

Clinical epidemiology is generally considered to be the basic science of clinical medicine [17], indicating its critical position in both medical education and the development of evidence-based medicine. A cohort study [18] indicated that epidemiology courses were also beneficial to the development of science literacy skills. As the study and application of epidemiology may continue throughout high school, undergraduate, graduate, doctoral and even working stages, the best practices in improving student learning enthusiasm and leading them to master the basic knowledge and practical skills of clinical epidemiology has become a tremendous challenge for educators.

Educational researchers have conducted numerous experiments to develop more feasible and effective teaching methods in epidemiology education. Dyke et al. [19] found that compared to traditional lectures, problem-based learning (PBL) formats can better mobilize students' learning enthusiasm, and a separate study demonstrated that students perform more actively when taught with the case-discussion method [20]. A flipped classroom model was applied in the “practice of epidemiology” course for third-year medical undergraduates, and study results showed its feasibility for future curriculum teaching reform [21]. The aforementioned studies were all aimed at transforming the traditional teaching approach into a student-oriented methodology and improving the learning enthusiasm of students during the learning process. Nevertheless, some students still see the learning of epidemiology as dry and boring, as the theoretical knowledge is often too abstract to understand, and these students report a desire for greater exposure to practical teaching, which is entirely consistent with the results of a previous study conducted in the UK [22]. Moreover, with the advent of the digital era and threats from COVID-19, remote learning is bound to become more prevalent. Although distance education has the advantages of decreased commuting time and a flexible schedule, it yet creates an inevitable barrier to conduct practical and clinically relevant education. Balancing student needs and social circumstances in this context is a challenge for educators.

In this study, we aimed to fully understand the needs of students and to apply practical and clinical courses to the epidemiological instruction of undergraduate medical interns. Through this course, students in the experimental group participated in conducting a cohort study and learned to deal with problems arising in the study process, which instructs students in the flexible application of theoretical knowledge and clinical scientific research. After the course, students in the experimental group had significantly higher survey scores in course understanding and clinical practice skills, while no significant difference was found in the control group, indicating that the additional practical course helped students deepen their comprehension of the course content (*p* < 0.0001) and make better use of epidemiological methods (*p* = 0.0001). Notably, the survey results from before the course showed that the self-rated scores in the control group were significantly higher than those in the experimental group regarding course content comprehension (*p* = 0.0060) and the utilization of research methods (*p* = 0.0019). These differences no longer existed after the completion of the course, which indicates the positive influence of this community-based course. In addition, the post-course survey results also revealed that students in the experimental group reported greater interest in the course (*p* = 0.0015), mastered the course more easily (*p* = 0.0167) and were more willing to participate in the course (*p* = 0.0113). Overall, the increase in self-evaluated scores indicated that taking the practical course could improve students' learning enthusiasm, interest and participation and develop their clinical practice skills through problem-solving and clinical research.

At the end of the course, more than 65% of students in both groups agreed that they have improved their understanding of clinical epidemiological information. Over 90% of the students admitted that this course was very rewarding and substantively helpful for them. The percentage of students who disagreed increased from 12.50% to 26.83% in the experimental group and from 35.29% to 55.88% in the control group, which demonstrated that some students were still unable to keep pace with the teaching content. There are two possible reasons for 17.65% of the students in the control group to express reluctance to participate in practical courses. One is that they may not be interested in practical course design and implementation, as they have taken epidemiology classes before, and the other is that they may be more accustomed to passive and exam-centred traditional methods, which is likely the main reason why 6.25% of students in the experimental group were dissatisfied with the course.

Although the results of follow-up tests showed that there were no significant differences between the scores of the two groups and the pass rate in both the theoretical part and the case analysis part, we still considered the practical course to have had a positive effect on strengthening the learning initiative, course understanding and clinical application of epidemiological knowledge and skills. Because the tests only covered a small portion of the knowledge points and the capability of practical application cannot be accurately evaluated simply with single choice questions even if several of those questions relate to actual case analysis.

Generally, students who participated in this two-week practical course exhibited a higher level of interest and learning enthusiasm, as well as a better mastery and utilization of epidemiological knowledge and skills.

This study also has some limitations. First, we only recruited undergraduate medical students who had enrolled in internships with The Fourth Affiliated Hospital of Zhejiang University in this study and over 70% of students came from Zhejiang University. Thus, the sample size was insufficient, and the results in this study may be unable to be generalized to all medical interns regarding practical courses. Moreover, the participants were randomly divided into the experimental group and the control group before the pre-course survey. However, the experimental group manifested higher interests than the control group before the study, which may lead to stronger learning motivation in the experimental group and result in biased outcomes. The role of the practical course may not be explained since significant difference in the baseline interests. Finally, the practical course allowed students to participate in all steps of clinical epidemiology research, but the research method used in this project was fixed (cohort study). This may familiarize students with cohort studies but leave them without an understanding of or capacity with other research methods, such as case‒control studies and cross-sectional studies. To better reveal the actual level of mastery and application ability of epidemiological knowledge and clinical skills, the further long-term study is needed. There is still a long way to go building developing and testing a suitable teaching evaluation system and confirming the effectiveness of its application.

In conclusion, offering a practical course in epidemiology to satisfy the needs of students is necessary, and this study showed that such practical courses are a beneficial supplement to the traditional teaching method, and it is conducive to strengthening students' learning enthusiasm and clinical practice ability as well as improving scientific research and clinical decision-making in general. Our study provides a new method and novel evidence for the development of an epidemiological teaching mode.

### Supplementary Information

**Additional file 1: Appendix Supplementary Table 1. **Responses to self-assessment survey of each group before and after taking epidemiological courses. **Supplementary Table 2.** The association between test score and the community practical course.

## Data Availability

The datasets used and analyzed during the current study are available from the corresponding author on reasonable request.
