# Supplementary material for: An effectiveness evaluation of a community-based course for medical students: a randomized controlled trial in the teaching of epidemiology
Source: BMC Med Educ. 2023 Oct 27;23:807. doi: 10.1186/s12909-023-04787-z (PMC10604501; doi:10.1186/s12909-023-04787-z)
Supplement: Supplementary file 1 — Additional file 1: Appendix Supplementary Table 1. Responses to self-assessment survey of each group before and after taking epidemiological courses. Supplementary Table 2. The association between test score and the community practical course. [file 12909_2023_4787_MOESM1_ESM.docx]

**Appendix Supplementary Table 1 Responses to self-assessment survey of each group before and after taking epidemiological courses**

| Survey number and questions | | Pre-course | | | | | | Post-course | | | | | |
| --- | --- | --- | --- | --- | --- | --- | --- | --- | --- | --- | --- | --- | --- |
|  |  | Experimental group (n=40) | | | control group (n=34) | | | Experimental group (n=41) | | | control group (n=34) | | |
|  |  | SD/D (%) | U (%) | SA/A (%) | SD/D (%) | U (%) | SA/A (%) | SD/D (%) | U (%) | SA/A (%) | SD/D (%) | U (%) | SA/A (%) |
| Q1 | I am interested in this course | 5.00 | 30.00 | 65.00 | 20.59 | 41.18 | 38.24 | 4.88 | 12.20 | 82.93 | 11.76 | 50.00 | 38.24 |
| Q2 | I truly want to participate in this practical course | 5.00 | 22.50 | 72.50 | 8.82 | 44.12 | 47.06 | 4.88 | 14.63 | 80.49 | 17.65 | 35.29 | 47.06 |
| Q3 | I am looking forwards to/satisfied with this course | 0 | 32.50 | 67.50 | 0 | 26.67 | 73.33 | 6.25 | 18.75 | 75.00 | 0 | 14.29 | 85.71 |
| Q4 | I comprehend the epidemiological course contents | 45.00 | 40.00 | 15.00 | 17.65 | 44.12 | 38.24 | 12.20 | 21.95 | 65.85 | 14.71 | 14.71 | 70.59 |
| Q5 | I think this course is easy to master | 12.50 | 57.50 | 30.00 | 35.29 | 52.94 | 11.76 | 26.83 | 34.15 | 39.02 | 55.88 | 23.53 | 20.59 |
| Q6 | I think practice is more helpful than theory in the teaching of this course | 5.00 | 27.50 | 67.50 | 5.88 | 44.12 | 50.00 | 7.32 | 26.83 | 65.85 | 5.88 | 23.53 | 70.59 |
| Q7 | I think that this course is helpful to medical scientific research | 0 | 2.50 | 97.50 | 0 | 8.82 | 91.18 | 0 | 4.88 | 95.12 | 0 | 8.82 | 91.18 |
| Q8 | I am good at utilizing epidemiological methods | 67.50 | 30.00 | 2.50 | 38.24 | 44.12 | 17.65 | 34.15 | 46.34 | 19.51 | 47.06 | 35.29 | 17.65 |
| Q9 | I think it is necessary for clinicians to be trained in this course | 0 | 20.00 | 80.00 | 2.94 | 11.76 | 85.29 | 0 | 4.88 | 95.12 | 2.94 | 11.76 | 85.29 |
| Q10 | I think it is helpful for clinicians to master epidemiological skills | 0 | 12.50 | 87.50 | 2.94 | 8.82 | 88.24 | 0 | 4.88 | 95.12 | 0 | 5.88 | 94.12 |

Values are given in percentages of medical interns; SD=Strongly disagree; D=Disagree; U=Uncertain; A=Agree; SA=Strongly agree.

**Supplementary Table 2 The association between test score and the community practical course**

|  |  | Model 0 | | Model 1 | | Model 2 | |
| --- | --- | --- | --- | --- | --- | --- | --- |
|  |  | coefficient β (95% CI) | *p* value | coefficient β (95% CI) | *p* value | coefficient β (95% CI) | *p* value |
| Theoretical part | Control | (Reference) |  | (Reference) |  | (Reference) |  |
|  | Experimental | 1.672 (-1.153, 4.496) | 0.250 | 1.944 (-0.935, 4.823) | 0.190 | 1.948 (-0.946, 4.843) | 0.191 |
| Case analysis part | Control | (Reference) |  | (Reference) |  | (Reference) |  |
|  | Experimental | 1.449 (-1.937, 4.834) | 0.404 | 1.109 (-2.339, 4.558) | 0.530 | 1.108 (-2.364, 4.580) | 0.534 |
| Total score | Control | (Reference) |  | (Reference) |  | (Reference) |  |
|  | Experimental | 3.120 (-1.187, 7.428) | 0.160 | 3.053 (-1.365, 7.472) | 0.180 | 3.056 (1.393, 7.505) | 0.182 |
|  |  | Model 3 | | Model 4 | | Model 5 | |
|  |  | coefficient β (95% CI) | *p* value | coefficient β (95% CI) | *p* value | coefficient β (95% CI) | *p* value |
| Theoretical part | Control | (Reference) |  | (Reference) |  | (Reference) |  |
|  | Experimental | 1.660 (-1.684, 5.004) | 0.334 | 1.747 (-1.394, 4.889) | 0.280 | 2.750 (-0.428, 5.928) | 0.095 |
| Case analysis part | Control | (Reference) |  | (Reference) |  | (Reference) |  |
|  | Experimental | 1.394 (-2.750, 5.538) | 0.512 | 0.485 (-3.327, 4.296) | 0.804 | -0.196 (-3.962, 3.571) | 0.919 |
| Total score | Control | (Reference) |  | (Reference) |  | (Reference) |  |
|  | Experimental | 3.054 (-2.203, 8.311) | 0.259 | 2.232 (-2.727, 7.191) | 0.381 | 2.555 (-2.460, 7.569) | 0.322 |

Test scores of students in the control group was set as the reference and test scores included scores of theoretical part, scores of case analysis part and total score. Model 1 adjusted for age; Model 2 adjusted for age and gender; Model 3 adjusted for age, gender and the perception dimension before the course; Model 4 adjusted for age, gender and the course understanding dimension before the course; Model 5 adjusted for age, gender and the clinical practice competency dimension before the course.
